# Enhancing the Mechanical Behaviour and Antibacterial Activity of Bioepoxy Using Hybrid Nanoparticles for Dental Applications

**DOI:** 10.1155/2022/2124070

**Published:** 2022-03-31

**Authors:** Mohammed Razzaq Mohammed, Ahmed Namah Hadi

**Affiliations:** ^1^Department of Mechanical Engineering, College of Engineering, University of Misan, Amarah, Iraq; ^2^Department of Biomedical Engineering, College of Engineering, University of Babylon, Hillah, Iraq

## Abstract

The appropriate capability of handling several forces exerted inside the mouth, and preventing the adhesion and proliferation of oral microorganisms are among the most vital factors for achieving effective alternative dental materials to the damaged native. Nevertheless, lack of mechanical and antimicrobial properties of dental resins hinders their use in most clinical applications in dentistry. In the present study, the main aim was to provide bioepoxy composite biomaterials that could meet the required mechanical and antibacterial properties for dental related fields. Herein, highly biocompatible epoxy and hybrid reinforcing materials were utilised to produce a composite material, which could have features resembling those of original dental parts. Various weight fractions of nanosilver/nano-alumina particles at 1, 2, and 3 wt% were incorporated into the bioepoxy for improving the mechanical and antibacterial characteristics of the biocompatible epoxy resin. Three-point bending and Izod impact tests were performed to evaluate the flexure and impact strengths of the obtained nanocomposites. The morphology of pristine bioepoxy and nanoparticle reinforced bioepoxy composites was characterized by scanning electron microscopy. The influence of these fillers on the bioepoxy resin antibacterial sensitivity was assessed using the agar diffusion technique. Nanofiller contents have been revealed to have a remarkable role to play in tuning the mechanical properties of the nanocomposites; the flexure strength and modulus were higher when the total ratio of hybrid reinforcement was 2 wt%. In contrast, the addition of higher percentage of hybrid nanoparticles could cause deterioration in the flexure characteristics of nanocomposites, yet they were better than those of pristine epoxy. Regarding the impact strength, the enhancement in this property was only observed for the composite containing 1 wt% of AgNps-Al_2_O_3_; the impact strength was dropped gradually beyond this ratio. The antibacterial effectiveness of the nanocomposites was demonstrated to positively depend on the increase in AgNps mass fraction. Among all evaluated unmodified and modified bioepoxy, the nanocomposite containing 2.5 wt% of AgNps had the higher antibacterial activity against *Escherichia coli* and *Staphylococcus aureus*. Based on the attainable outcomes, the prepared composites, particularly at moderate levels of Al_2_O_3_-AgNps, could provide biocomposites having the potential to be utilised in several biomedical fields, particularly in dental technology.

## 1. Introduction

The use of resins in dental applications are varied, including restorative materials, cavity liners, crowns, denture teeth, provisional restorations, root canal posts, and structured scaffolds [1, 2]. Thermosetting resins provide biocompatibility, a suitable environment for the part used inside the mouth, aesthetic qualities and reasonable cost, making these polymers preferred materials in various dental applications [3–5]. The polymeric composites used in these applications consist of an organic material, which represents the matrix phase. The most frequently used polymers in this field are methacrylates, epoxy and polyethylene [6, 7]. They are strengthened with a reinforcing phase, commonly inorganic dispersed materials, as these additives not only enhance aesthetic, optical and mechanical properties but also decrease curing shrinkage [8].

Epoxy is regarded as one of the supreme thermosets that have been applied as a matrix in composites owing to its great stiffness and strength, chemical resistance, and ease of processing. Furthermore, epoxy resins have low cure shrinkage [9], and various curing agents such as amines can be utilised in order for the polymerisation and creating a network of the epoxy [10]. Therefore, based on the presence of multiple chemicals used in the formation of epoxy, in addition to the flexibility to use different manufacturing approaches for fabricating epoxy-based products, the applications of this resin have become highly varied in many areas, including forming composites. Among the diverse kinds of epoxy resins, the epoxy of diglycidyl ether of bisphenol-A (DGEBA) has appropriate mechanical and biocompatible properties to be used in a variety of biomedical fields such as orthopaedic and dental related applications [11, 12].

In the course of the last few decades, nanocomposites have become a significant class of materials with desirable features [13]. Polymer-based nanocomposites possess the unique capability to have enhanced physical and mechanical characteristics at low reinforcement content, thereby being lighter than those of conventional micro-scaled composites [14, 15]. To improve the performance of the biocomposite, a variety of materials have been used as a reinforcing phase within the composites. Silver nanoparticles (AgNps) are the most regularly used nanoparticles in numerous applications as a result of their ductility, electrical and thermal conductivity, and antimicrobial activity even at low concentrations against several kinds of microorganisms [16, 17]. Such nanoparticles have presented antimicrobial impacts on several microbes including *E. coli and Candida albicans* [18, 19]. Besides, contrasting to antibiotics, the antibacterial effect of AgNps is not limited to one specific site in the bacteria, but includes several levels including bacterial wall and deoxyribonucleic acid (DNA) [20]. AgNps have the ability to alter the structure of bacteria cell membranes and even causing cell death. The efficacy of these nanoparticles is attributable to their size and large ratio of surface area to volume [21]. Various studies have been conducted recently using AgNPs to fabricate composites for dental related applications [22]. Findings obtained from these studies on the use of this type of composite were not identical. It was found that the incorporation of AgNps reduced the flexural strength of PMMA-based composites in comparison with the filler-free polymers [23]; moreover, the addition of AgNps decreases the tensile strength of composites [24]. Nevertheless, it was indicated from another study that the strength of the PMMA-AgNps composite was improved compared to the AgNps-free polymer [25, 26]. The impact of AgNps on the flexural strength of PMMA was revealed to rely on a number of aspects including the polymer kind and the nano fillers loading [27]. In terms of biological activity, the presence of AgNps within polymer exhibited no detrimental impact on cellular activity toward several kinds of cells [28].

Alumina (Al_2_O_3_) has also been paid tremendous attention as a candidate for an extensive range of biomedical applications owing to its appropriate mechanical characteristics and bio-inertness [29]. In dentistry, the impact of inclusion Al_2_O_3_ into the polymer matrix was examined in a number of studies for enhancing the mechanical properties of the matrix [30–32].

Despite the improvement in the mechanical and antibacterial properties of dentistry materials, a wide variety of dental applications require further enhancement. The object of this project was to examine the mechanical and antibacterial characteristics of bioepoxy loaded by hybrid AgNps/Al2O3 nanoparticles to be potentially utilised for dental technology. This particular type of epoxy was chosen to be the matrix in this study where it has been proven to be biocompatible. Using different kinds of reinforcements can generate new features for the resulting composite in which the matrix or mono-filler composite could not have. The presence of reasonable-price alumina, on the one hand, can lead to enhancement in the rigidity of the epoxy that has brittle behaviour. On the other hand, AgNps can improve the antibacterial activity of the resin even better as these nanofillers are considered among the leading antimicrobial nanoparticulate metals. Nano-sized reinforcements have been used, for such materials provide a high surface area and a rise in the possibility of bonding particles with the resin; consequently, increasing the mechanical properties of the formed composite compared to the microsized composites. Thus, the presence of this hybrid reinforcement system may provide a suitable environment to improve not only the mechanical performance of the bioepoxy, but may also contribute in reducing or preventing bacterial growth.

## 2. Materials and Methods

### 2.1. Materials

Silver and alumina nanoparticles with an average size of 60 nm and 50 nm respectively, were obtained from Nanjing Emperor Nano Material CO., LTD (China). The DGEBA epoxy and isophoronediamine (IPD) hardener were purchased from Hangzhou Dely Technology Co., Ltd (China).

### 2.2. Preparation of Epoxy/Al2O3-AgNps Nanocomposites

Epoxy resin and AgNps-Al_2_O_3_ nanoparticles of different concentrations of 0.5, 1.5, and 2.5 wt% of AgNps and at constant loading of 0.5 wt% Al_2_O_3_ were mixed. The mixture of hybrid nanoparticles and bioepoxy resin were subjected to mechanical mixing for 10 minutes in order for the nanoparticles to be homogenously dispersed within the resin. After adding IPD hardener to the blend at 2 : 1 resin: hardener, the obtained mixture was thoroughly stirred for 3 minutes to form the nanocomposites. Samples for the flexural and impact tests were prepared by pouring the epoxy composites into a silicon mould and finally cured for 10 hours at room temperature.

### 2.3. Scanning Electron Microscopy (SEM)

Microscopic observations of neat bioepoxy and bioepoxy reinforced by AgNps and Al_2_O_3_ nanoparticles were conducted using scanning electron microscopy (SEM) INSPECT F 50 FE-SEM apparatus. Samples were coated with a 50 nm thick gold film prior to SEM tests.

### 2.4. Flexure Test

Pristine bioepoxy and bioepoxy that was filled with nanoparticles of Al_2_O_3_-Ag were tested for flexure strength and modulus using three-point bending. This was conducted according to ASTM D790 by means of Zwick universal testing machine.

### 2.5. Izod Impact Test

The impact tests were conducted on the base of ISO-180 standard using XJU series pendulum Izod impact testing at an impact velocity of 3.5 m/s. Unnotched Izod impact was used to measure the material impact strength that was assessed from the pendulum kinetic energy for specimens prepared at dimensions of 80 × 10 × 4 mm.

### 2.6. Antibacterial Activity

Disc diffusion method was applied for evaluating the antibacterial activities of *E. coli* and *S. mutans* that were cultured in Muller-Hinton broth. The bacteria were activated and leaved in the incubator for 24 hours at 37°C prior to dilution. Small pieces of neat bioepoxy and bioepoxy containing 0.5 wt% AgNPs-0.5 wt% Al_2_O_3_ and 2.5 wt% AgNPs-0.5 wt% and Al_2_O_3_ were formed and exposed to the inhibition zone tests. Following sterilizing, the specimens were placed on *E. coli* and *S. mutans* culture plates. The agar plates were incubated for a whole day at physiological temperature. The relative antibacterial consequence was established by assessing the zones of inhibition produced around the specimens.

### 2.7. Statistical Analysis

GraphPad Prism 9 was employed for data statistical analysis. The mean and standard deviations (SD) of the data were calculated. Mechanical properties data were analysed using a one-way ANOVA, with *p* < 0.05 representing a significant difference between means. The data pertaining to antimicrobial activity of *Escherichia coli* and *Staphylococcus aureus* were also analysed with two-way ANOVAs by a Turkey multiple range test to assess the influence of the increasing of the loading nanofiller within the nanocomposites on antimicrobial activity.

## 3. Results and Discussion

### 3.1. Nanocomposite Morphology

**Figure 1:**
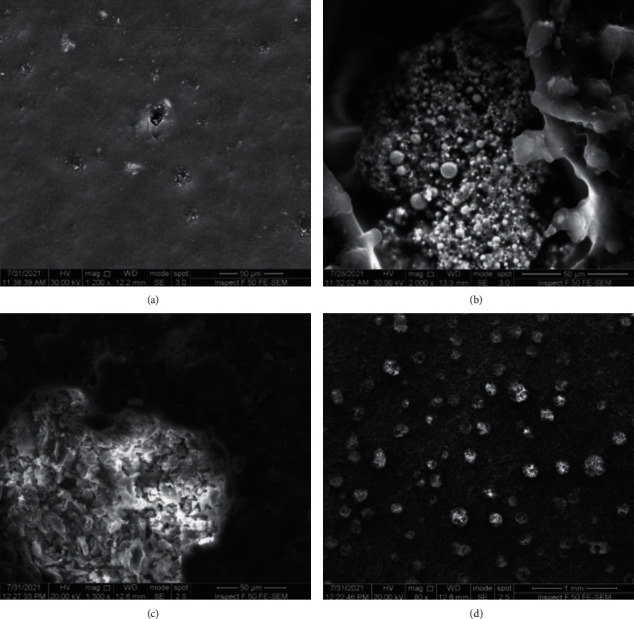
SEM micrographs of (a) bare bioepoxy (b) bioepoxy/1wt% Al2O3-AgNps naocomposite, and (c, d) bioepoxy/3wt% Al2O3-AgNps nanocomposite at various magnifications. Scale bar (a-c) = 50 *µ*m, (d) = 1 mm.

The morphology of the bioepoxy was changed notably after modification with Al_2_O_3_-AgNps. While the blank bioepoxy exhibited a smooth flat surface (Figure 1(a)), the presence of the nanofillers can be clearly noticed after the reinforcement (Figures 1(b)–1(d)). The hybrid mixture of nanoparticles was observed to be well dispersed within the nanocomposites. It was also appeared that some agglomerations of nanoparticles were occurred in the nanocomposites, particularly at high filler weight fraction, resulting in micropores. Such micropores could be created owing to bioepoxy monomer evaporation when its temperature increased during the polymerisation process. These results are in agreement with the finding that acquired by Karthic et al. [33]; they noticed that micropores were generated in PMMA-based composites containing high loading of nano sea shell powder, causing dropping in the values of microhardness of the composites.

### 3.2. Flexural Strength and Modulus

**Figure 2:**
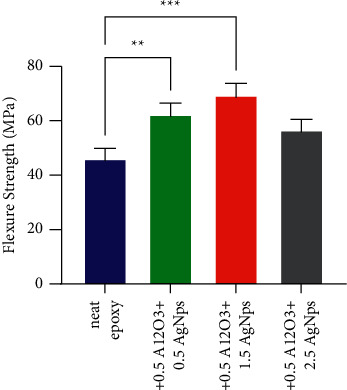
Flexure strength for pristine bioepoxy and bioepoxy reinforced by various weight fractions of Al_2_O_3_-AgNps. Asterisks indicate that results significantly different to the unmodified control (^*∗∗*^*p* < 0.01; ^*∗∗∗*^*p* < 0.001).

**Figure 3:**
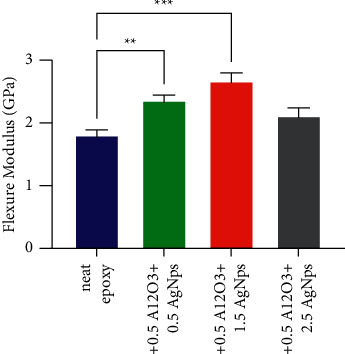
Flexure modulus for pristine bioepoxy and bioepoxy reinforced by various weight fractions of Al_2_O_3_-AgNps. Asterisks indicate that results significantly different to the unmodified control (^*∗∗*^*p* < 0.01; ^*∗∗∗*^*p* < 0.001).

Flexural properties of bare bioepoxy and bioepoxy nanocomposites filled with AgNps-Al_2_O_3_ at various mass fractions were examined using three-point bending test. Figures 2 and 3 illustrate the impact of incorporating hybrid nanofillers consisting of Al_2_O_3_ at a fixed weight fraction of 0.5 wt% and different ratios of AgNps ranging between 0.5 and 2.5 wt% on the flexural strength and flexural modulus. In general, it was noticed occurring a major enhancement in the values of flexure strength and modulus after including a mixture of reinforcement materials. For the flexure strength, this increase was 35 and 60% at a ratio of 1 and 2 wt% of hybrid filler, respectively. Regarding the flexure modulus, a similar behaviour occurred; the values of this property gradually improved with an increase of up to 58% for the composite containing 3 wt% of Al_2_O_3_-AgNps compared to nanoparticle-free bioepoxy. Statistical analysis of data illustrated in Figures 2 and 3 indicate that flexure properties of bioepoxy reinforcing by 1 and 2 wt% of hybrid nanoparticles were significantly increased compared to those of the untreated bioepoxy. Such rise in both the flexure strength and modulus at low content of nanofiller are due to the chain mobility restriction. Moreover, the presence of very small size nanoparticles, which provide a large surface area increases the surface bonding and interaction between the fillers and the resin, providing high flexural strength. Besides, the presence of these nanoparticles offers a suitable environment for transferring and distributing the load and stress between the matrix and the reinforcement phase, where its effectiveness improves as the reinforcement percentage increases, resulting in improving the mechanical properties of the composites. Beyond 2 wt%, the quality of distribution played a profound role in decreasing flexure strength and modulus as the resulted composites had poor nanoparticles dispersion within the matrix leading to generating clusters of particles in the bioepoxy matrix. Van der Waal forces between the particles could be increased at high particle contents, which may lead to minimal dispersion of such nanoparticles. Consequently, a great level of reinforcement caused a reduction in the interaction quality between the matrix and reinforcement phase, resulting in ineffective load transfer within the composite system and inadequate mechanical properties.

### 3.3. Impact Strength

**Figure 4:**
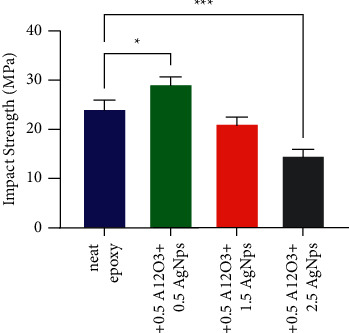
Impact strength for pristine bioepoxy and bioepoxy reinforced by various weight fractions of Al_2_O_3_-AgNps. Asterisks indicate that results significantly different to the unmodified control (^*∗*^*p* < 0.05; ^*∗∗∗*^*p* < 0.001).

The impact strength of the pristine and bioepoxy-based composite was evaluated using Izod test.  Figure 4 displays the impact strength of the nanocomposites as a function of AgNps-Al_2_O_3_ loading. The dispersion and interaction of the filler particles within the matrix play a deep role in determining the fracture energy of the nanocomposites. Due to the addition of 1 wt% of hybrid nanoparticles, a significant enhancement on the impact behaviour of the nanocomposite was accomplished. On the other hand, beyond this ratio, the Izod energy gradually decreased; the impact strength of nanocomposites containing 2wt% of the hybrid filler decreased with no significant difference from the control group (blank bioepoxy) (*p* > 0.05). Conversely, 3 wt% AgNps-Al2O3-based composite showed a significant decrease in its impact strength. At a low loading of 1wt% of hybrid fillers, the proper distribution of hybrid filler within the composite made an enhancement in impact properties to be more obvious than those for nnaocomposites containing higher amounts of fillers. Furthermore, increasing the mass fraction of AgNps on account of Al_2_O_3_ could cause the composite material to lose some ductility that Al_2_O_3_ may provide. The impact strength is a measure of the amount of energy needed to break the sample at a sudden and rapid load. Therefore, as it can be noticed when the material is brittle, as in the case of unmodified epoxy, the amount of energy requisite to break the sample is small. On the contrary, when the resin was strengthened with a higher ductility material namely, alumina, the composite material became able to absorb more energy and consequently improved impact strength. In addition, the presence of these ductile nanoparticles provided a suitable environment regarding the transferring and distribution of the stress generated on the matrix into the reinforcing material as a result of the strong bonding existed between the polymer and the ceramic-metal hybrid reinforcement. At high nanoparticle loading, this behaviour was different; the reinforcing material could be considered as sites of stress concentration, which means that these nanofillers of a great weakness to the composite. Subsequently, the composite reinforced with a high percentage of nanoparticles was sensitive to rapid and sudden forces, and those samples failed with low impact energy.

### 3.4. Anti-bacterial Activity Results

**Figure 5:**
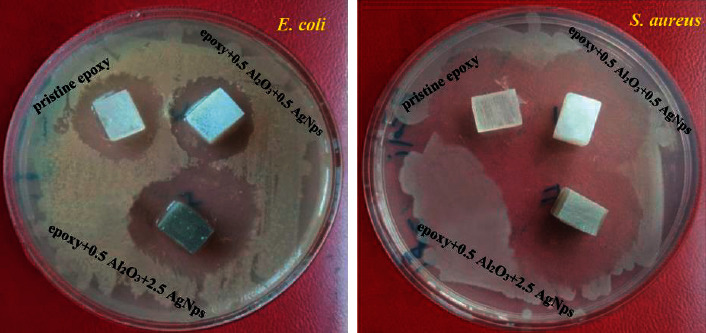
Zone of inhabitation of pristine bioepoxy and bioepoxy reinforced by various weight fractions of Al_2_O_3_-AgNps against *E. coli* and *S. aureus*.

**Figure 6:**
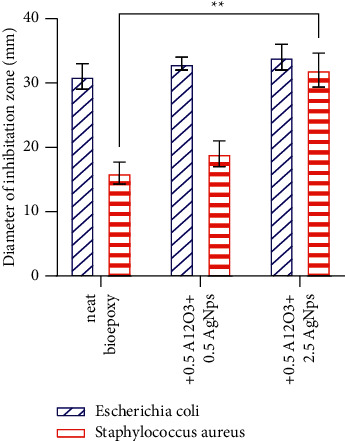
Antibacterial activity of pristine bioepoxy and bioepoxy reinforced by various weight fractions of Al_2_O_3_-AgNps against *E. coli* and *S. aureus*. Asterisks indicate that results significantly different to the unmodified control (^*∗∗*^*p* < 0.01).

Various kinds of microorganisms such as bacteria can be grown inside the mouth. *E. coli* (Gram negative) and *S. aureus* (Gram positive) bacteria were used to evaluate the antibacterial characteristics of neat bioepoxy and bioepoxy-based nanocomposites. It was pointed out that the nanosized particles could be more effective against bacteria adhesion and growth owing to the large surface area of these fillers [34]. Findings of bactiostatic rate revealed in Figures 5 and 6 exhibit the positive effect of AgNps on the two types of bacteria. The effectiveness of nanocomposites against *E. coli* enhanced after AgNPs incorporation at 1.5 and 2.5 wt% by around 6% and 10%, respectively. Likewise, this composite system was demonstrated to be efficient against *S. aureus* growth as a function of AgNPs loading; the inhibition rate improved to about 18% and 100% for the composites containing 1.5 and 2.5 wt% of AgNPs, respectively. Analysis of total bacterial growth by 2-way ANOVA revealed that inhibition zone was not significantly increased and was significantly increased for *E. coli* and *S. aureus*, respectively with increasing AgNps content. In other words, the sensitivity of *S. aureus* growth along with AgNPs increment was more obvious than that of *E. coli* even though the later was generally inhibited more efficiently. These outcomes are in line with previous studies that have revealed the capability of AgNps to destroy bacteria even at low concentrations [35], with no severe toxic impacts on human cells [36]. Bacterial inhibition was directly related to AgNPs mass fraction, yet the particular response of each of these kinds was determined by their metabolic features [37]. Ag ions that can be regularly released from AgNPs could kill microorganisms [38]. In consequence of electrostatic attraction, Ag ions can adhere to the cell wall and cytoplasmic membrane [39]; once the uptake of ions into cells, respiratory enzymes can be deactivated, and the formation of adenosine triphosphate can be interrupted [40]. Besides, reactive oxygen species are generated which they can result in the inducement of cell membrane disruption and DNA alteration. Ag ions can not only lead to negative issues in DNA replication and cell reproduction but can also impede the synthesis of proteins by denaturing ribosomes in the cytoplasm [41]. AgNPs are also capable of permeating bacterial cell walls and afterwards tune the structure of the cell membrane, and can themselves kill bacteria where the accumulated AgNPs in the pits that create on the cell wall can cause cell membrane denaturation [42]. *E. coli* membranes display negative electrostatic charges, promoting AgNPs diffusion in which these nanoparticles could permeate through the *E. coli* strains membrane without difficulty and interact with the protein, triggering configurational alterations and death [43]. AgNPs have also been stated to minimize adhesion ability and prevent growth of *S. aureus* [44]. Compared to Gram positive strains, Gram negative strains were strongly affected by all batches used whether the material was modified or not. The cell wall structure in Gram positive bacteria has a thicker peptidoglycan membrane, which could result in diminishing diffusion and reducing the antimicrobial efficiency [43, 45, 46].

This study examined the development and characterization of novel hybrid nanoparticle-bioepoxy composite system for potential use in dentistry. Remarkable enhancements both in mechanical and antimicrobial features have been obtained with the fabricated composites. However, it is considered that using coupling agents can be even more beneficial for increasing the correlation between nanofillers and the resin, contributing in much improvement in the mechanical performance of the composites. Besides, more work regarding the cytotoxicity of this composite system is required. Therefore, these issues can be taken into the consideration for further work to ensure the suitability of this composite system for use in medical arena, especially in dental applications.

## 4. Conclusions

Having dental related issues may not only cause oral health problems, but may also negatively affect several places in the body and thus pose a threat to the patient's life. A set of properties should be available for the materials to be used in dentistry, including having proper mechanical properties, hindering the adhesion and growth of microorganisms, giving an aesthetic appearance similar to the original part, and being available at affordable price. Herein, bioepoxy was reinforced with various weight fractions of AgNps and Al_2_O_3_. The morphology of bioepoxy was tuned considerably after modification, and the dispersion of the nanoparticles relied remarkably on the nanoparticle content. The flexural modulus and strength assessing for Al_2_O_3_-AgNps/bioepoxy composites were positively affected, mainly for the composite containing 2 wt% of hybrid nanoparticles. Nonetheless, with the addition of more content of AgNps, the flexural strength and modulus as well as the impact strength reduced which may be attributable to the agglomeration of the nanofillers and the poor adhesion of the nanofillers and the matrix. The influence of these nanoparticles on the bioepoxy antibacterial activity were being encouraging where the results could be interpreted by the ability of nanoparticles to effect on both Gram negative and Gram positive ability to be adhered and ingrowth onto the obtained nanocomposites. Based on these findings, the resulting composite system can be a proper candidate for various clinical applications in dentistry such as for prosthetic, restorative, endodontic, orthodontic, and implant treatment.

## Data Availability

The research data used to support the findings of this study are included within the article.
